# Supplementary material for: Green Product Types Modulate Green Consumption in the Gain and Loss Framings: An Event-Related Potential Study
Source: Int J Environ Res Public Health. 2022 Aug 29;19(17):10746. doi: 10.3390/ijerph191710746 (PMC9517842; doi:10.3390/ijerph191710746)
Supplement: Supplementary file 1 [file ijerph-19-10746-s001.zip › ijerph-1829768-supplementary.pdf]

Supplementary Materials

1.1 Manipulation of Green products

30 questionnaires were collected on the Wenjuanxing website (https://www.wjx.cn/) to conduct pre-experimental evaluation of eighteen types of green products. The participants were asked to rate whether the green product is more beneficial to themselves or the environment according to the product attributes on the Likert 9 points scale (ranging from “1” = very beneficial to themselves; to “9” = very beneficial to the environment). Taking 5 as the median value, a single-sample *t*-test was performed on the average scores of eighteen green products. The results showed that the participants thought that they were more beneficial to themselves when evaluating nine self-interested green products, and when they evaluated nine kinds of other-interested green products, they thought they were more beneficial to the environment (see Table A1). This showed that the green products we chosen could well represent the type they belong to. Then, 30 questionnaires were collected again on the Wenjuanxing website to evaluate the familiarity, arousal, product preference of green products with a 9-point scale. Using independent samples *t*-test, it was found that self-interested green products and other-interested green products were not significantly different in familiarity (Mself-interested = 6.06 ± 1.30, Mother-interested = 5.50 ± 1.20, t (58) = 1.709, p = 0.093), arousal (Mself-interested = 5.89 ± 1.16, Mother-interested = 5.73 ± 0.80, t (58) = 0.632, p = 0.530), and product preferences (Mself-interested = 6.48 ± 1.10, Mother-interested = 6.28 ± 1.04, t (58) = 0.724, p = 0.472) were not significantly different.

Table S1. Pre-experimental evaluation of green product types

**Green product type**	**Products Name**	***M*±*SD***	***t***
Self-interested	1 Natural botanical shower gel	3.90±2.14	-2.817**
	2 Organic apple	3.43±2.36	-3.638***
	3 Natural vegetable soap	3.80±2.17	-3.026**
	4 Organic vegetables	3.00±2.17	-5.058***
	5 Natural organic cotton clothing	2.67±1.73	-7.393***
	6 Green rice	2.77±2.16	-5.662***
	7 Pure plant natural toothpaste	3.07±2.10	-5.043***
	8 Green wheat bread	3.10±1.94	-5.375***
	9 Natural organic cotton sheets	3.57±2.42	-3.249**
Other-interested	1 Recycled pulp flower pot	7.87±0.97	16.134***
	2 Eco-friendly gift box	7.13±1.46	8.026***
	3 Degradable plastic cup	8.47±0.78	24.466***
	4 Recycled paper	6.77±2.11	4.581***
	5 Degradable garbage bags	8.13±0.97	17.635***
	6 Environmentally friendly mobile phone holder	6.70±1.84	5.057***
	7 Degradable eco-friendly paper towels	7.53±1.41	9.857***
	8 Recycled environmental umbrellas	7.17±1.56	7.629***
	9 Green battery	7.60±1.57	9.089***

Note：* p<0.05，** p<0.01，*** p<0.001

1.2 Manipulation check of Message Framings

The effectiveness of the message framing manipulation was tested and 30 questionnaires were randomly distributed and collected on the website (https://www.credamo.com/#/), including 18 female and 12 male (aged between 20 and 36, with a mean age of 27.23 years). Participants were asked to judge whether the sentence described "the purchase (not buying) of environmentally friendly products would benefit you (the environment) or would it cost you (the environment)", "the purchase (not buying) of the environmentally friendly products would bring you (the environment) a positive outcome or a negative outcome" and rate their feelings after reading on the Likert 7 points scale (ranging from "1" = very benefit (very positive); to "7" = very loss (very negative))(Baek & Yoon, 2017).

To examine the effectiveness of the message framing, the one-sample *t*-test was performed on the gain framing and the loss framing with 4 as the median value. The results showed that when the participants evaluated the gain framing ($M \pm SD$ = 1.64 ± 0.67), they believed that buying green products would benefit themselves (the environment) or bring a positive outcome (t (29) = -19.42, $p < 0.001$, Cohen's d = -7.21). In the same way, when they evaluated the loss framing (5.93 ± 0.77), they believed that purchasing green products would cause themselves (the environment) loss or bring a negative outcome (t (29) = 13.78, $p < 0.001$, Cohen's d = 5.12). These results suggested that the manipulation of the gain framing and the loss framing was effective, and these two sentences could effectively induce the participants' gain and loss feelings.

1.3 Behavioral results

In order to exclude the effect of price level on the main variable, a repeated measure analysis of variance (ANOVA) of 2 (green product type: self-interested vs. other-interested) × 2 (message framing: gain vs. loss) × 7 (price level: 25% vs. 50% vs. 75% vs. 100% vs. 125% vs. 150% vs. 175%) was conducted for the total proportion of purchasing green products.

Table S2. Proportion of purchasing green products in purchasing decisions under four conditions (*M* ± *SD*)

		**Total proportion of purchasing green products (%)**	
**Price Level**	**Message Framing**	**Self-interested**	**Other-interested**
25%	Gain framing	61.91 ± 28.96	59.40 ± 31.53
	Loss framing	65.40 ± 34.15	61.94 ± 32.47
50%	Gain framing	49.83 ± 30.56	45.63 ± 29.11
	Loss framing	57.74 ± 35.22	50.77 ± 33.15
75%	Gain framing	40.23 ± 29.29	37.03 ± 27.70
	Loss framing	44.77 ± 34.55	43.71 ± 32.77
100%	Gain framing	31.94 ± 29.79	32.00 ± 28.82
	Loss framing	41.54 ± 34.72	35.14 ± 29.64
125%	Gain framing	28.20 ± 31.20	29.09 ± 27.26
	Loss framing	35.80 ± 34.25	36.43 ± 32.24
150%	Gain framing	24.06 ± 30.83	26.86 ± 27.80
	Loss framing	31.00 ± 33.76	31.03 ± 33.24
175%	Gain framing	22.80 ± 32.24	23.66 ± 26.11
	Loss framing	29.74 ± 33.99	30.06 ± 30.95

There were significant main effects for price level (F (6, 204) = 39.05, $p < 0.001$, $\eta_p^2 = 0.54$), and message framing (F (1, 34) = 4.40, $p = 0.043$, η2 p = 0.12). After multiple comparisons, it was found that, as shown in Table A2, as the price of green

products increased, the proportion of participants who purchased green products decreased (*ps* < 0.001). When it was 150% higher, there was no significant difference in the proportion of participants who purchased green products caused by a price difference that was 175% higher than that of common products ($p = 0.085$).

The interaction of green product and message framing was not significant, F (1, 34) = 0.52, $p = 0.475$, $\eta_p^2 = 0.02$. The interaction between green product type and price level was not significant, F (6, 204) = 2.36, $p = 0.065$, $\eta_p^2 = 0.07$. The interaction between message framing and price level was not significant, F (6, 204) = 0.61, $p = 0.620$, $\eta_p^2 = 0.02$. The triple interaction of green product type, message framing and price level was also not significant, F (6, 204) = 0.67, $p = 0.635$, $\eta_p^2 = 0.02$ (see Figure A1). These results showed that the price level in the paradigm did not affect our main variables.

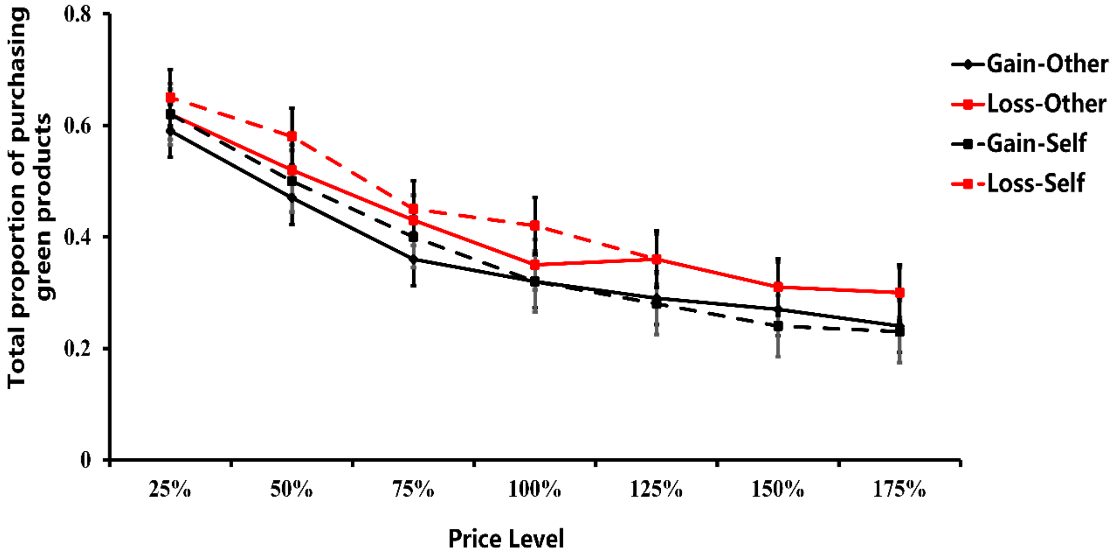

Figure S1. Line graph of the total proportion of green product purchases in each condition. Gain-self: gain framing-self-interested green products, Loss-self: loss framing-self-interested green products, Gain-other: gain framing-other-interested green products, Loss-other: loss framing-other-interested green products.

1.4 ERP results

According to the waveform results, N1 component and LPP component were found. However, there was no difference between the self- and other-interested green products under the gain or loss framing about two components. Therefore, their results were only reported in the appendix.

The N1 was defined as the most negative peak in the period of 80–135 ms and the LPP was measured as the mean amplitude within the time window of 420–650 ms. Based on the topographical distribution of each ERP component and previous literature (Zubair et al., 2020), N1 statistics was calculated across six electrode sites in the fronto-central region (F3, Fz, F4, FC3, FCz, and FC4), whereas LPP statistics were reported across 10 electrode sites in the central-parietal region (CP1, CP3, CPz, CP2, CP4, P1, P3, Pz, P2, and P4). Mean amplitude values were averaged for all selected electrode sites. All data were statistically analyzed using SPSS 26.0. The N1 and LPP amplitude were each analyzed using a two-way repeated-measures analysis of variance (ANOVA) of 2 (green product types: self-interested vs. other-interested) × 2 (message framing: gain vs. loss). The significance level for all analyses was set at 0.05. Post hoc comparisons were Bonferroni-corrected at $p < 0.05$. The Greenhouse-Geisser correction was conducted to account for sphericity violations whenever appropriate, and the partial eta-squared (η_p^2) was reported as a measure of effect size.

1.4.1 N1

A repeated-measures ANOVA of the N1 amplitude revealed that both main effects and interactions were not significant (all p values > 0.05).

1.4.2 LPP

There was no other main effect on the LPP amplitude. A repeated-measures ANOVA of the LPP amplitude revealed a marginally significant interaction effect, F (1, 34) = 3.73, $p = 0.062$, $\eta_p^2 = 0.10$. The simple effect analysis revealed that the LPP was marginally larger for self- (1.35 ± 0.27μV) than for other-interested green products (0.81 ± 0.34μV) in the context of the gain framing, F (1,34) = 2.99, $p = 0.093$, but in the context of the loss framing, the LPP showed no difference between self-interested green products (1.34 ± 0.29μV) and other-interested green products (1.55 ± 0.34μV), F (1,34) = 0.46, $p = 0.501$.

Reference:

Baek, T. H., & Yoon, S. (2017). Guilt and Shame: Environmental Message Framing Effects. *Journal of Advertising, 46*(3), 440-453. doi:10.1080/00913367.2017.1321069

Zubair, M., Iqbal, S., Usman, S. M., Awais, M., Wang, R., & Wang, X. (2020). Message framing and self-conscious emotions help to understand pro-environment consumer purchase intention: an ERP study. *Scientific Reports, 10*(1), 1-8. doi:10.1038/s41598-020-75343-8
